# A Proving of Oleum-animale

**Published:** 1896-10

**Authors:** C. L. Olds

**Affiliations:** Philadelphia, Pa.


### A PROVING OF OLEUM-ANIMALE.

C. L. OLDS, M. D., PHILADELPHIA, PA.

On November 10th, 1894, at 11 A. M., took Oleum-animale $^{50m}$ (Swan), one dose dry on the tongue.

November 14th, 9 A. M.—Sensation as if sore throat would come on—for a short time only.

November 15th, 11 A. M.—Throat sore on left side; aggravated by empty swallowing; ameliorated while eating or drinking; causes much hawking and swallowing; has not had a sore throat or cold for several years; much mucus in throat; eyes ache.

November 16th.—Throat very sore and raw in centre; aggravated by empty swallowing; ameliorated by food and liquids; much white, frothy mucus in; constant desire for food.

November 16th, 5 P. M.—Thirsty; throbbing headache, aggravated by motion.

November 17th, forenoon.—Sore throat disappeared in the night; headache, dull, aggravated by shaking head; drawing, pressive pain at root of nose; increased sexual desire (mental); lascivious thoughts. Afternoon—Headache, throbbing, involving only right side of head and right eye, aggravated by lying down and stooping; slight cough; dripping of watery, excoriating mucus from left nostril when in a warm room, ameliorated in open air; much sneezing.

November 18th.—Large quantities of thick, yellow mucus detached from low in throat with difficulty; head stopped up in house, clear in open air; chilly in house, but cold symptoms, ameliorated in open air; lips feel very dry, sensation of white of egg dried on them.

November 19th, 7 A. M.—Large quantities of yellowish mucus detached from throat. 8.30 P. M.—Headache, right side only, involving right eye; pains dull, pressive from occiput to right eye; symptoms of the cold about gone.

November 20th.—Small boil, not very sore, but itchy, on inner aspect of left thigh.

November 23d, 8 P. M.—Mucous membrane of buccal cavity seems much relaxed; can hardly keep from biting it when eating; aching in legs, as though cold was coming on.

November 25th, 8.30 A. M.—Throbbing, lancinating pain in internal right ear for a few minutes.

December 1st.—Soreness and burning in spots of roof of mouth; easily irritated for several days; appetite normal during forenoon, but in afternoon, no matter how large a dinner he ate, has almost constant craving for food—no kind in particular; eats an enormous dinner, and then eats something about every fifteen or thirty minutes during afternoon.

December 2d, 3 P. M.—Sharp, knife-like pain, starting in left side of occiput and going through into left eye—for a short time only. 8 P. M.—Under part of tongue, left side, sore as after taking too hot drink.

December 4th, 10.30 A. M.—Sharp, itching pains in lower hypochondrium and left lumbar region at every deep breath—for a few minutes only. 8.10 P. M.—Sore, drawing pain extending from right external abdominal ring down spermatic cord to right testicle on walking; testicle felt as if pain would cease if it was suspended.

December 5th.—Dull aching in small of back in morning before rising, and at 10 A. M. while sitting, passing away on walking.

December 8th, 11 A. M.—Took Oleum-animale $^{cm}$ (F.), one dose dry.

December 10th.—Little appetite for breakfast; during entire afternoon burning sensation in stomach; burning eructations; load in stomach as if food did not digest. 9 P. M.—Sharp, knife-like pains in right side of occiput.

December 12th.—Restless at night.

December 13th.—Restless at night; irritable; sharp, piercing pain over left eye, off and on; dripping of a yellowish water from the nose, especially on stooping.

December 17th.—Restless at night; dreamed that he saw a man drown but was not at all concerned, but watched him go down with scientific interest only; irritable, slightest thing makes angry; dull, aching pain in back; aching-drawing in both groins.

December 21st.—Restless nights; dull aching in thighs and legs, better by motion; restless, feels as if he must move or keep working rapidly; easily irritated; ejaculation too soon during coition.

December 30th.—Dreamed that he was being tried for murder, and later that the executioners were preparing to hang him, but a great army of mighty snakes so terrified them that they ran away; woke with aching in small of back; aching continued nearly all day; dull and stupid in morning; worse in morning; better afternoon and evening.

### SYSTEMATIC ARRANGEMENT OF SYMPTOMS.

MIND.—Lascivious thoughts; irritable; slightest thing makes him angry.

HEAD.—Throbbing headache, aggravated by motion; dull headache, aggravated by shaking head; throbbing headache, involving only right side of head and right eye, aggravated by stooping or lying down; dull, pressive pains going from right occiput to right eye; sharp, knife-like pains starting in left occiput and going to left eye; sharp, knife-like pains in right side of occiput; sharp, piercing pain over right eye.

EYES.—Aching in eyes.

EARS.—Throbbing, lancinating pain in internal right ear.

NOSE.—Drawing, pressive pain at root of nose; much sneezing; dripping of a yellowish water from the nose, especially on stooping; dripping of watery, excoriating mucus from left nostril when in warm room, ameliorated in open air; catarrhal symptoms, ameliorated in open air.

MOUTH.—Lips feel very dry; sensation of white of egg dried on them; mucous membrane of buccal cavity seems much relaxed, can hardly keep from biting it when eating; soreness and burning in spots of roof of mouth; under part of tongue, left side, sore, as after taking too hot drink.

THROAT.—Sore throat, left side; causes much hawking and swallowing; aggravated by empty swallowing; ameliorated while eating or drinking, with much thirst and desire to eat constantly; much white, frothy mucus in throat; profuse, thick yellow mucus, detached with difficulty.

APPETITE, THIRST.—Thirsty for cold drinks; almost constant craving for food in afternoon, although he ate heartily at dinner; eating or drinking relieves the sore throat; poor appetite for breakfast.

STOMACH.—Burning sensation in stomach; burning eructations; feeling of load in stomach as if food did not digest.

ABDOMEN.—Sharp, stitching pains in left hypochondrium and left lumbar region at every deep breath; aching-drawing in both groins.

SEXUAL ORGANS.—Increased sexual desire, with lascivious thoughts; ejaculation too soon during coition; sore, drawing pain extending from right external abdominal ring down spermatic cord to right testicle on walking; testicle felt as if pain would cease if it was suspended.

COUGH.—Cough better in open air.

BACK.—Dull aching in small of back in morning before rising, and at 10 A. M. while sitting, passing away on walking.

LIMBS.—Aching in lower extremities, as if cold was coming on; dull aching in thighs and legs, better by motion; small, itching boil on inner aspect of left thigh.

REST, MOTION.—Restless at night; restless, must move or keep working rapidly.

SLEEP, DREAMS.—Restless sleep; dreams of seeing man drown, but was not concerned, watched him with scientific interest only; of being tried for murder; of being executed; of snakes.

GENERALITIES.—Worse in the morning; better afternoon and evening; in open air; after eating.

